# Perspective on the origin, resistance, and spread of the emerging human fungal pathogen *Candida auris*

**DOI:** 10.1371/journal.ppat.1011190

**Published:** 2023-03-23

**Authors:** Cheshta Sharma, David Kadosh

**Affiliations:** Department of Microbiology, Immunology and Molecular Genetics, University of Texas Health Science Center at San Antonio, San Antonio, Texas, United States of America; University of Maryland, Baltimore, UNITED STATES

## What explains the origin, emergence, and persistence of *Candida auris*?

*Candida auris* is a major emerging human fungal pathogen that was first reported in 2009 as an isolate from the ear canal of a patient in Japan [1]. Since this initial report, *C*. *auris* has grown to represent a “serious threat” in healthcare settings, as indicated by the United States Centers for Disease Control (CDC) and is now on the World Health Organization (WHO) fungal priority pathogen list, described as the “most wanted” critical pathogen [2,3]. However, many questions remain about the emergence, spread, and persistence of *C*. *auris*. In particular, the sudden, simultaneous, and independent worldwide emergence of 5 *C*. *auris* clades in completely separate geographical regions is a profound puzzle.

One hypothesis suggests that *C*. *auris* was not detected until recently as a consequence of nonreliance on conventional phenotypic typing methods. Correct identification of *C*. *auris* is crucial for the adequate treatment and control of outbreaks and has been a frequent limitation. *C*. *auris* can be grown under similar conditions to those of other *Candida* species and single colonies can be obtained on conventional Sabouraud dextrose agar following 24 hours of incubation at 30 to 35°C [4]. However, the ability of *C*. *auris* to grow at temperatures up to 42°C differentiates it from other *Candida* species [5]. In clinical microbiology laboratories *C*. *auris* is frequently undetected, as 90% of isolates are misdiagnosed as *Candida haemulonii*, *Candida famata*, *Candida guilliermondii*, *Candida lusitaniae*, *Candida parapsilosis*, *Candida sake*, *Rhodotorula glutinis*, *Candida duobushaemulonii*, *Candida catenulata*, *Candida tropicalis*, or *Saccharomyces cerevisiae*, with commercial identification systems that utilize biochemical phenotyping [6–8].

*C*. *auris* appears white, pink, or purple on conventional CHROMagar *Candida* chromogenic medium (Becton-Dickinson, Rungis, France) but on CAN2 plates (bioMérieux, Capronne, France), colonies are initially white, but later appear as a light reddish pink color [5]. Recently, CHROMagar *Candida* Plus (Becton-Dickinson, Rungis, France) and HiCrome *C*. *auris* MDR selective agar (HiMedia, Mumbai, India) have been found to be highly specific and sensitive for the isolation and identification of *C*. *auris* after 36 to 48 hours of incubation [9–11]. FDA-approved methods such as MALDI-TOF mass spectrometry, combined with up-to-date spectra databases, as well as the user-made MSI library (Paris, France) have also been used for the definitive identification of *C*. *auris* [12]. Finally, a combination of D1/D2 and ITS sequencing have proven to be the gold standard for *C*. *auris* identification [13]. While recent advances in fungal molecular diagnostics have improved *C*. *auris* detection and identification, the hypothesis that *C*. *auris* was not accurately detected is not alone sufficient to explain the sudden emergence of this species, since a reanalysis of *Candida* isolates between 1997 and 2016 detected only a small number of misidentified *C*. *auris* strains [14].

Another hypothesis suggests that extensive use of antifungals in the clinic and in agricultural settings could serve as a possible selective force in generating a transmission reservoir of antifungal-resistant *C*. *auris* strains; a similar hypothesis has been used to explain the emergence of azole-resistant *Aspergillus fumigatus* strains [15]. In support of this hypothesis, several *C*. *auris* strains, most of which were cross-resistant to medical and agricultural azoles, have been isolated from the surface of apples, although contamination from human handling cannot be excluded [16]. However, this hypothesis cannot explain the sudden worldwide emergence of *C*. *auris* belonging to 5 different clades, which suggests that other important contributing factors play a role.

The recently described “global warming emergence hypothesis” suggests that an increase in global warming led to the simultaneous emergence of thermal tolerant *C*. *auris* in different geographical locations [17]. Because *C*. *auris* is tolerant of temperature and salinity, Casadevall and colleagues have proposed that prior to being recognized as a human pathogenic species, *C*. *auris* existed as a plant saprophyte in specialized ecosystems, such as wetlands [18]. In support of this hypothesis, Arora and colleagues recently described the first environmental isolations of *C*. *auris* from a sandy beach and a salt marsh wetland in the Andaman Islands, India [19]. The isolation of 2 clonal strains, one of which exhibited slow growth at 37° and 42°C and was susceptible to antifungals and a second that grew well at 37° and 42°C, suggested a close association with the wild *C*. *auris* inhabiting the environment. These findings suggest that *C*. *auris* existed as a slow growing and drug-susceptible pathogen, which acquired thermal tolerance initially as a consequence of global warming and then developed drug resistance after its adaptation in humans [13]. In addition, the fact that *C*. *auris* colonizes colder body areas and is unable to grow in the absence of free oxygen may suggest an environmental origin. Acquisition of virulence traits in *C*. *auris* could also be explained by a combination of global warming and UV radiation that might have induced genetic mutations and/or epigenetic changes leading to improved adaptability for growth in different ecological niches [18,20]. Although the global warming hypothesis is well supported and may seem attractive, other factors, including global human migration, poor hygiene, and high population densities, should not be ignored and might also have contributed to the development of persistence and antifungal resistance in *C*. *auris*.

## What are the molecular and genetic determinants of antifungal resistance in *C*. *auris*?

*C*. *auris* is best known for its strong resistance to a wide variety of antifungal therapies. Based on the tentative breakpoints proposed by the CDC, about 90%, 30%, and 2% to 10% of *C*. *auris* isolates are resistant to the major antifungal drugs fluconazole (FLU), amphotericin B (AMB), and echinocandins, respectively [21]. Overall, about 90% of *C*. *auris* strains have acquired resistance to at least 1 drug, 30% to 41% are resistant to 2 drugs and about 4% are resistant to all 3 antifungals [21]. It is also important to mention here that *C*. *auris* has been classified in 5 phylogenetically distinct clades based on whole-genome sequence data, with each clade differing from the other clades by >200,000 SNPs [22–25]. Table 1 describes the main characteristics of each clade. Recently, genomic and centromeric analyses have also demonstrated extensive rearrangements across all 7 chromosomes of *C*. *auris* [23,26,27]. The broad geographical distribution and genetic diversity among *C*. *auris* isolates belonging to different clades, as well as conflicting virulence reports, suggest that all clades need to be studied extensively and that the data from one clade cannot necessarily be extrapolated to others.

**Table 1:** Main characteristics of *Candida auris* clades.

Characteristics	Clades				
	South Asian (I)	East Asian (II)	African (III)	South American (IV)	Iranian (V)
**Antifungal susceptibility profile**	Resistant to FLU, cross-resistant to echinocandins and AMB, some are pan-resistant	Lower resistance to antifungal agents	Resistant to FLU, cross-resistant to echinocandins and AMB, some are pan-resistant	Resistant to FLU, cross-resistant to echinocandins and AMB, some are pan-resistant	Resistant to FLU, cross-resistant to echinocandins and AMB, some are pan-resistant
**Clinical isolation site**	Ear, blood, or other invasive sites	Mainly ear	Ear, urine, blood, or other invasive sites	Blood, or other invasive sites	Nail, skin, ear
**Mating type**	MTLa	MTLα	MTLα	MTLa	Not known
***ERG11* mutations**	Y132F or K143F	K143R, L43H, Q357K	F126L	Y132F, K143R, K177R, N335S, E343D	Y132F, I466L
***TAC1B* mutations**	R495G, A640V, A657V, A15T, S195C, P595L	F214S	None	F214S, F862_N866del, K247E, M653V, A651T, P595H	Not known
**Outbreaks**	Invasive infections	Ear infections	Invasive infections	Invasive infections	Invasive infections
**Geography**	Dominates in the United States, Europe, South Asia	Dominates in Korea, Japan	Dominates in Europe, Africa	Dominates in the United States	Dominates in Iran
**Phenotypes**					
Growth on actidione	No	Yes	Yes	Not known	Not known
Pseudohyphae	Yes	No	No	Not known	Not known
Large cellular aggregates	No	Yes	Yes	Not known	Not known
Assimilation of L-rhamnose	No	No	Yes	No	Yes
Utilization of N-acetyl glucosamine	Yes	No	Yes	Yes	Yes

AMB, amphotericin B; del, deletion; FLU, fluconazole.

Data obtained from references [4,13,24,25,28–30].

Azole resistance in *C*. *auris* has been mainly attributed to mutations in the *ERG11* gene, which encodes the azole target lanosterol 14-α-demethylase (Table 2) [30,31]. Increased *ERG11* copy number, largely observed in Clade III strains, may amplify the effects of these mutations. This finding also suggests clade-specific variation in azole resistance mechanisms [31]. Increased expression of *CDR1*, an ABC-transporter, has been shown to substantially contribute to *C*. *auris* clinical azole resistance and significantly increased expression of *MDR1*, encoding a MFS transporter, has been observed in FLU-resistant Clade III clinical *C*. *auris* strains (Table 2) [32–34]. Mutations in several transcriptional regulators that control the expression of drug efflux pumps, including *TAC1B* and *MRR1A*, have also been associated with altered *C*. *auris* antifungal susceptibility (Tables 1 and 2) [35–37]. Whole-genome comparison of *C*. *auris* to other sequenced *Candida* species has revealed the notable expansion of gene families linked to virulence and drug resistance, including siderophore-based iron transporters, secreted lipases, and oligopeptide transporters [23].

**Table 2:** Mechanisms of antifungal resistance in *Candida auris*.

	Amino acid substitutions (if applicable)	Impact on MIC (if known)	Clades	Mechanism of action (if known)	References
**Triazole resistance**					
**Gene mutations**				leads to the formation of 4, 4-dimethyl cholesta-8, 14, 24-trienol, instead of ergosterol	
*ERG11*	VF125AL	8–16-fold increase	III		[22,31,38,39]
	K143R	8–16-fold increase	I (subclade c), II, IV		[22,31,38,39]
	Y132F	8–16-fold increase	I (subclade b), IV		[22,31,38,39]
	F444L	4-fold increase	IV		[40]
	L43H, Q357K, G459S		II, IV		[38]
	I466M*, Y501H*	No impact on FLU, VRC MIC			[41]
*TAC1B*	A640V	16-fold increase	I (subclade c)	controls *CDR1* expression	[35,37]
	S611P	4-fold increase	IV		[40]
	A657V		I (subclade b)		[35,37]
	F862, N866del		IV		[35,37]
	R495G^		I		[35,37]
	F214S^		II, IV		[35]
	S192N, A583S		I, II		[35]
	A15T, S195C		I		[35]
	P595L		I		[35]
	P595H		IV		[35]
	K247E, A651T, M653V		IV		[35]
*MRR1A*	N647T	4-fold increase in FLU, VRC MICs	III, IV	controls *MDR1* expression	[34,36,42]
**Gene deletions**					
*TAC1B*		2–4-fold decrease FLU MICs; 4–8-fold decrease in VRC MICs	III, IV	controls *CDR1* expression	[36]
*CDR1*		64–128-fold decrease in FLU MICs	I	increases drug efflux	[32,33]
**Aneuploidy/copy number variations**					
Increased *ERG11* copy number (2–3 copies)	Y132F K143R VF125AL K143R, Y132F		I II III IV		[37,44,45,47] [46,47] [43,47] [46,47]
**Echinocandin resistance**					
***FKS1* mutations**					
Hot spot 1	S639F/Y		I, III	decreases the sensitivity of β-(1,3)-D-glucan synthase to drug	[39,48–51]
	S639P		IV		[52]
	F635del, F635L/Y, S639T, D642Y		I (subclade c)		[53–56]
Hot spot 2	R1354S	6-fold increase			[50]
**AMB resistance**					
**Gene mutations**					
*ERG6*	YY98V*	≥32-fold increase	I (subclade b)	leads to the generation of cholesta-type sterol instead of ergosterol	[56]
*FLO8*	utg5_821828 (C/T)				[57]
**Gene overexpression**					
*ERG1*, *ERG2*, *ERG6*, *ERG13*					[23]
**5-Flucytosine resistance**					
**Gene mutation**					
*FUR1*	F211I				[49]
**Terbinafine resistance**					
Upregulation of a *CDR6* ortholog					[58]

del, deletion; FLU, fluconazole; MIC, minimum inhibitory concentration; VRC, voriconazole.

* Tested by substitutions in *S*. *cerevisiae*.

^ Identified by in vitro evolution experiments.

In 2019, Ruiz and colleagues showed that the genome of *C*. *auris* undergoes substantial karyotypic reorganization under stress conditions [43]. The authors also hypothesized that as neither polyploid states nor sexual reproduction have been described in *C*. *auris*, it is highly likely that this species is not capable of generating genome diversity via aneuploidy or polyploidy. However, Burrack and colleagues have recently shown that *C*. *auris* acquires aneuploidy very rapidly in just 3 passages [44]. This aneuploidy can be easily detected in the population during in vitro evolution, in the presence and absence of FLU [44]. Another instance of rapid acquisition of FLU resistance in *C*. *auris* due to adaptive aneuploidy was demonstrated where an extra copy of chromosome V was gained in the presence, but lost in the absence, of FLU [45]. A similar in vitro acquisition of azole resistance in an evolving population derived from a single *C*. *auris* parent strain was explained by aneuploidy occurring in the form of segmental duplications, coexisting alongside aneuploidy-independent mechanisms [37]. In addition, Narayanan and colleagues recently demonstrated a novel mechanism of antifungal resistance involving generation of a mitotically stable supernumerary chromosome, which leads to an increase in the copy number of a set of genes in *C*. *auris* [46].

Echinocandins inhibit the activity of 1,3-β-D-glucan synthase, an enzyme encoded by the *FKS1* and *FKS2* genes, which is important for synthesis of a primary fungal cell wall polymer. About 2% to 10% of clinical *C*. *auris* strains exhibit echinocandin resistance, which usually emerges during treatment [13]. The most commonly observed mutations associated with echinocandin resistance in *C*. *auris* are located in the *FKS1* hot spot regions (Table 2), although their direct role in echinocandin susceptibility has not yet been assessed [39,48–56]. Interestingly, in a *C*. *auris* outbreak study, an isolate was found to be resistant to both echinocandins and 5-flucytosine (5-FC) [43]. Echinocandin resistance in this strain was associated with a serine to tyrosine amino acid substitution in the gene *FKS1*, while resistance to 5-FC was associated with a phenylalanine to isoleucine substitution in the gene *FUR1* (Table 2) [49].

AMB has broad-spectrum activity against pathogenic fungi and exerts its antifungal effect by directly binding to ergosterol. While AMB resistance is rare in fungi, there have been reports suggesting a higher prevalence (approximately 30%) of AMB resistance in clinical *C*. *auris* strains, based on tentative CDC breakpoints. Recent studies have shown that mutations in *ERG6* (encoding C-24 sterol methyltransferase) are associated with *C*. *auris* AMB resistance (Table 2) [56,59]. In vitro exposure of an AMB-resistant *C*. *auris* strain to AMB also resulted in increased expression of *ERG1*, *ERG2*, *ERG6*, and *ERG13* (Table 2) [23]. Wasi and colleagues analyzed changes in the transcript levels of *C*. *auris* ABC transporters by qRT-PCR after short-term exposure to AMB and terbinafine [58]. Interestingly, the *CDR6* ortholog CAUR_04233 exhibited greater than 8-fold higher expression compared to that of other ABC transporters in response to AMB. The *CDR6* ortholog CAUR_04233 was also strongly up-regulated following terbinafine treatment (Table 2) [58]. However, because the majority of the AMB-resistant strains lack mutations in ergosterol biosynthesis pathway genes, alternative resistance mechanisms are likely. Escandón and colleagues identified SNPs in AMB-resistant isolates in a transcription factor similar to *FLO8* in *Candida albicans* (Table 2) [57]. Although these findings do not provide definitive proof of involvement of these proteins in AMB resistance, they do provide a future direction for research into AMB resistance in *C*. *auris*.

For most human fungal pathogens, such as *C*. *albicans*, the level of antifungal resistance remains low. However, clinical outcomes are poor, leading to high mortality rates due to treatment failure [14]. The immune status of the patient, along with other host factors and pharmacologic issues, including interaction of drug and the fungus, can directly or indirectly affect therapeutic responses [60]. This contradictory relationship between low clinical resistance and an overall clinical outcome of therapeutic failure could be attributed to antifungal tolerance [61]. Antifungal tolerance/trailing growth is usually characteristic of susceptible strains that tend to grow slowly at inhibitory drug concentrations [62,63]. Azole tolerance in *C*. *auris* is enhanced as mother cells age, requires the molecular chaperone *HSP90*, and has been associated with gene duplication as well as overexpression of *ERG11* and *CDR1* [33,47]. Based on these studies, *C*. *auris* shows increased tolerance to azoles and could be more tolerant to echinocandins as well [33,47]. It is pertinent to mention here that *C*. *auris* in vitro evolution experiments have used MIC measurements to define antifungal resistance. However, information about the range of drug tolerance in clinical strains and how tolerance changes over time is still lacking [37,45]. Because the degree of tolerance and resistance varies depending on the intrinsic allele diversity of each isolate and phenotypic heterogeneity, it is important to understand the contribution of the genetic background of the species [63–65]. Recently, Burrack and colleagues evolved a set of 17 clinical isolates of *C*. *auris* belonging to different clades to determine whether resistance is stable in the absence of drug, the frequency of drug tolerance, and how genetic background affects strain evolutionary outcome [44]. Interestingly, they found that drug tolerance can occur in *C*. *auris* and shows variation among clinical isolates similar to that observed for other *Candida* species. This study also suggested that antifungal tolerance acquired by *C*. *auris* over time could ultimately lead to resistance [44,63,66].

Although antifungal resistance remains a key feature of most *C*. *auris* infections, relatively little is known about resistance mechanisms in *C*. *auris* compared to those of other human fungal pathogens and many important questions remain outstanding. How do resistance mechanisms differ among *C*. *auris* strains belonging to different genetic clades? Why is multidrug resistance more prevalent in *C*. *auris* compared to other pathogenic fungi? Are there completely novel mechanisms that make *C*. *auris* unique in its ability to tolerate antifungals? Future studies that address these questions are most likely to have the greatest impact on our understanding of *C*. *auris* drug resistance mechanisms.

## How did *C*. *auris* prevail during the COVID-19 pandemic?

Since its first report in late 2019, Severe Acute Respiratory Syndrome Coronavirus 2 (SARS-CoV-2), the virus responsible for Coronavirus Disease 2019 (COVID-19), has rapidly spread, causing a global health emergency that resulted in the official announcement of a massive pandemic by WHO in 2020 [67,68]. COVID-19 has exacerbated several preexisting conditions, leading to an increase in bacterial, fungal, and viral coinfections and superinfections in hospitalized patients [69]. Fungal infections pose a greater challenge, as a consequence of high mortality rates, limited diagnostics, and increased antifungal resistance, and severe clinical COVID-19 further increases the risk of invasive fungal infections [69]. *C*. *auris* is one such fungal pathogen that causes outbreaks in COVID-19 ICUs and hospitals worldwide [69]. *C*. *auris* patients shed viable yeast cells continuously from their skin, leading to the contamination of hospital environments, especially ICUs. Both *C*. *auris* and SARS-CoV-2 have been found in the hospital setting, including on IV poles, bedrails, hospital floors, windows, and air conditioner ducts [69]. COVID-19 patients become potentially more susceptible to *C*. *auris* colonization and infection as they develop acute respiratory distress syndrome requiring ICU admission, mechanical ventilation, and/or extracorporeal membrane oxygenation [69]. Importantly, *C*. *auris* and SARS CoV-2 share several common risk factors, including chronic kidney disease, diabetes mellitus, as well as the administration of broad-spectrum antibiotics and systemic steroids.

COVID-19-associated *C*. *auris* infections have been reported in over 10 countries, including Mexico, Brazil, Lebanon, India, Italy, Iran, Spain, Turkey, Greece, Pakistan, Qatar, Colombia, and the US [70–83]. An initial study from India reported a 60% case-fatality rate for COVID-19 patients, of which two-thirds had a *C*. *auris* coinfection [78]. Another study from Mexico showed a high mortality rate of 83% in COVID-19 patients with *C*. *auris* bloodstream infections [79]. A study from the US reported the isolation of 3 *C*. *auris* bloodstream infections and 1 urinary tract infection in 4 patients with COVID-19 [80]. Magnasco and colleagues screened 118 patients admitted to COVID-19 ICUs in Italy and found 5.1% (6 patients) to be colonized/infected with *C*. *auris*. Of these 6 patients, 4 developed *C*. *auris* candidemia [81]. Brazil also reported their first 2 cases of *C*. *auris* in December 2020, both in patients from the same COVID-19 ICU [82]. Similarly, Lebanon reported its first isolation of *C*. *auris* from 14 patients, all of whom were admitted to 4 separate critical care units [83]. Half of these patients were infected with COVID-19 prior to isolation of the *C*. *auris* [83].

While it is crucial to rapidly identify and isolate patients colonized with *C*. *auris*, the pandemic has made it challenging to do so, due to overburdened, resource-limited, and overwhelmed hospital settings. Unfortunately, the COVID-19 pandemic has provided ideal conditions for *C*. *auris* outbreaks in hospital ICUs. As a consequence, more effective antifungal therapies are in even greater demand.

## How to strategize therapeutics: New drugs or drug combinations?

Based on frequency of resistance profiles and the available literature, the echinocandin drug micafungin has been recommended as the first-line treatment for *C*. *auris* infections in adults [84]. However, the cost and limited availability of this treatment in most countries is a major concern. AMB is also recommended as a first-line treatment for neonates and infants [84].

Given the challenges of resistance, novel antifungals are needed in order to effectively treat *C*. *auris* infections. Several new antifungal agents with potential therapeutic effects against *C*. *auris* have been developed that are undergoing Phase II or Phase III clinical trials. Ibrexafungerp is the first representative of a novel class of glucan synthase inhibitors, known as triterpenoids, and is the most promising. It possesses potent activity against *C*. *auris* strains, including echinocandin-resistant isolates, in vitro leading to cellular deformation and pore formation, as well as inhibition of cell division [85]. Rezafungin is a novel long-lasting echinocandin that exhibits potent activity against *C*. *auris* isolates both in vitro and in vivo in a neutropenic mouse model [85]. However, this drug shows reduced activity against echinocandin-resistant *C*. *auris* isolates. Another new antifungal compound, MYC-053 (sodium 5-[1-(3,5-dichloro-2-hydroxyphenyl) methylideneamino]-6-methyl-1,2,3,4-tetrahydro-2,4-pyrimidinedionate), synthesized by TGV-Therapeutics (Wilmington, DE), is a representative of a novel class of antifungal agents [86]. MYC-053 functions by simultaneously inhibiting both intracellular nucleic acid synthesis and targeting the synthesis of chitin, a key cell wall component [86]. This small molecule compound shows strong fungicidal activity against azole- and echinocandin-resistant isolates and has potential activity against biofilms [86]. Fosmanogepix is another novel antifungal that targets the enzyme Gwt1, which is necessary for localization of phosphatidylinositol-anchored proteins to the fungal cell wall [85]. This drug exhibits potent activity against resistant *C*. *auris* isolates and has also shown strong in vivo efficacy in a murine-invasive candidiasis model. Finally, VT-1598 is a new tetrazole that selectively inhibits fungal Cyp51A (lanosterol demethylase). In a neutropenic mouse model of *C*. *auris* infection, treatment with VT-1598 resulted in a significant increase in survival and a reduction in both brain and kidney fungal burden [85].

Several additional potential therapeutics for the treatment of *C*. *auris* infections have been identified through high-throughput screening of drug repurposing libraries, including the organoselenium compound ebselen, alexidine dihydrochloride, and the antiparasitic drugs miltefosine and iodoquinol [87]. Given the number of therapeutics currently under development and in the pipeline, several of which could potentially be used in combination with current therapies, the probability of successfully treating life-threatening *C*. *auris* infections is likely to be significantly improved in the future.
